# Examining the Flexural Behavior of Thermoformed 3D-Printed Wrist–Hand Orthoses: Role of Material, Infill Density, and Wear Conditions

**DOI:** 10.3390/polym16162359

**Published:** 2024-08-20

**Authors:** Daniel Vlăsceanu, Diana Popescu, Florin Baciu, Constantin Stochioiu

**Affiliations:** 1 Department of Strength of Materials, Faculty of Industrial Engineering and Robotics, National University of Science and Technology Politehnica Bucharest, 060042 Bucharest, Romania; daniel.vlasceanu@upb.ro (D.V.); constantin.stochioiu@upb.ro (C.S.); 2 Department of Robotics and Production Systems, Faculty of Industrial Engineering and Robotics, National University of Science and Technology Politehnica Bucharest, 060042 Bucharest, Romania

**Keywords:** 3D printing, wrist–hand orthosis, thermoforming, PLA, PETG, infill density, mechanical properties, moisture, natural aging

## Abstract

This paper examined the mechanical properties of wrist–hand orthoses made from polylactic acid (PLA) and polyethylene terephthalate glycol (PETG), produced through material extrusion with infill densities of 55% and 80%. These orthoses, commonly prescribed for wrist injuries, were 3D-printed flat and subsequently thermoformed to fit the user’s hand. Experimental and numerical analyses assessed their mechanical resistance to flexion after typical wear conditions, including moisture and long-term aging, as well as their moldability. Digital Imaging Correlation investigations were performed on PLA and PETG specimens for determining the characteristics required for running numerical analysis of the mechanical behavior of the orthoses. The results indicated that even the orthoses with the lower infill density maintained suitable rigidity for wrist immobilization, despite a decrease in their mechanical properties after over one year of shelf life. PLA orthoses with 55% infill density failed at a mean load of 336 N (before aging) and 215 N (after aging), while PETG orthoses did not break during tests. Interestingly, PLA and PETG orthoses with 55% infill density were less influenced by aging compared to their 80% density counterparts. Additionally, moisture and aging affected the PLA orthoses more, with thermoforming, ongoing curing, and stress relaxation as possible explanations related to PETG behavior. Both materials proved viable for daily use, with PETG offering better flexural resistance but posing greater thermoforming challenges.

## 1. Introduction

Additive manufacturing (also known as 3D printing—3DP) is currently used in many domains [1,2,3], as it offers important advantages over the traditional manufacturing technologies in respect to personalization, design freedom, on-demand and delocalized production, and a shorter supply chain [4], therefore positively impacting the whole product life cycle. This manufacturing technology satisfies the modern market requirements of mass customization and resource efficiency [5], provides competitive benefits [6], and opens up new opportunities, such as the implementation of 3D printing point-of-care (3DP-PoC) [7,8,9]. Bringing the manufacturing closer to the point of need is considered beneficial across different medical realms, including orthopedics [10], cranio-maxillofacial surgery [11], or personal protective equipment production [12]. For achieving the aforementioned advantages of 3DP-PoC, and for extending the range of applications towards other fields like orthotics and prosthetics, efficient digital workflows are necessary, as well as reliable engineering information about specific aspects related to material selection, 3DP process parameters settings, mechanical behavior of the product, impact of aging, moisture, etc.

A growing interest in the use of 3DP technology for obtaining orthoses/splints, as well as the advantages and the current barriers to a wider spread, are noted in the recent literature [13,14,15]. Wrist–hand orthoses (WHOs) are commonly encountered, as wrist–related injuries have a high worldwide incidence [16], clinical studies on 3DP-WHOs showing promising results [17,18]. However, there are also drawbacks mentioned in the reviewed studies and in practice. One relates to the design process, which requires engineers with 3D modeling skills. Another drawback is the long printing times of the 3DP-WHOs in their end-use form, which is usually based on 3D scanning the patient’s limb. Therefore, thermoforming a flat-shaped orthosis to fit the user’s forearm was proposed as an alternative [19]. This way, the printing time is reduced while the design knowledge is captured in customizable templates based on discrete measurements obtained by the therapists without the designers’ support. Additionally, compared to the vertically built 3DP-WHOs (in their end-use form), flexible sensors can be more easily embedded into thermoformed orthoses [20,21], for instance. Table 1 presents a comparative SWOT diagram for both approaches, 3D scan-based and thermoforming-based, emphasizing the most significant characteristics.

In the context, this research investigated the influence of two materials (PLA—polylactic acid and PETG—polyethylene terephthalate glycol) and two infill densities (55% and 80%) on the 3D-printed volar WHO’s flexural behavior corresponding to flexion movements similar to lifting a weight. Both experimental and numerical investigations were conducted with the purpose of providing a methodology to investigate the strength of the orthoses without performing real tests for every design. As the rationale for this study was to gather relevant engineering data on the performance of 3DP-WHOs under conditions specific to everyday use, the effects of natural aging and moisture on the flexural properties were experimentally studied, given that the literature completely lacks information in this regard. Moreover, the literature also revealed that there are a few studies that have focused on testing 3DP-WHO’s mechanical resistance [22], increasing the amount of data in this respect being necessary for gaining doctors’ and patients’ confidence in these devices with slim designs [23].

Thermoforming is a manufacturing process that shapes plastic sheets by heating them above their glass transition temperature (Tg) and then cooling them into a rigid structure or object. PLA and PETG are two popular materials for the MEX process, and they can also be subjected to thermoforming at relatively low temperatures, hence their selection for this study. PLA has a melting point of 160–190 °C and a Tg in the 55–65 °C range. It softens and can be deformed with manual force. PETG has a higher melting point of 220–260 °C and a Tg around 85 °C and is known for its flexibility, durability, and resistance to moisture and chemicals. PLA is commonly used as feedstock for 3D-printed orthoses [24], and recent studies have shown that PETG is also a feasible material that can withstand the stresses induced by real-world situations at an appropriate material thickness [25].

Both PLA and PETG are influenced by moisture and aging, these degradation conditions leading to changes in their mechanical properties. PLA and PETG 3D-printed specimens subjected to moisture were reported to become brittle [26,27], with PLA being affected due to its organic composition, while research on aging has shown that exposure to ultraviolet radiation (UV) can cause modifications in the stress response and mechanical properties for both PLA and PETG [26,28,29]. For instance, UVB radiation can reduce the tensile and compressive strengths of PLA and PETG parts, with PETG being more significantly affected [28]. Considering that real-world situations cause the orthoses to come into contact with warm water and to be worn over various periods of time, the current study aims to answer the following question: How do material selection and infill density influence the flexural properties and long-term usability of 3D-printed orthoses under typical use conditions, including the impacts of natural aging and water exposure?

This study main novelty is that it provides an experimental and numerical investigation of the flexural resistance of two 3DP-WHOs from two thermoformable materials under typical wear conditions. It also highlights the potential advantages of thermoforming flat-printed orthoses in reducing the production time and costs while maintaining a suitable rigidity for wrist immobilization. Additionally, the thermoforming approach offers a viable alternative to the other manufacturing methods, with potential for improved efficiency and scalability. The findings on the long-term usability and durability of the materials under real-world conditions further contribute to addressing gaps in the literature regarding the mechanical resistance of 3D-printed WHOs.

## 2. Materials and Methods

### 2.1. WHOs and Specimens Design and 3D Printing

**Figure 1:**
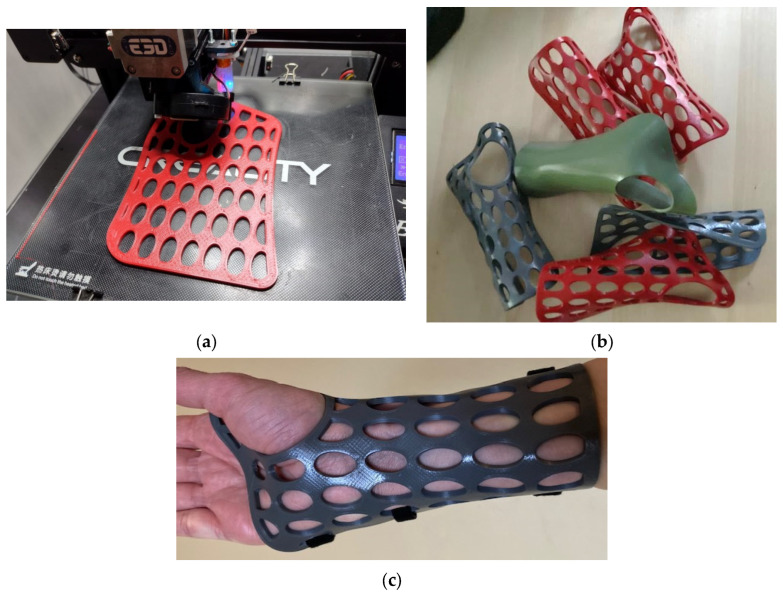
Three-dimensional printing wrist–hand orthoses: (**a**) example of a flat orthosis from PETG during the printing process; (**b**) examples of different thermoformed orthoses from both PLA and PETG; and (**c**) a PLA orthosis placed on the user’s hand and secured with three Velcro strips in the palm, wrist, and forearm zones.

Flat-shaped WHO models with a thickness of 2.7 mm were generated (Figure 1) based on the key dimensions measured on a healthy person’s forearm and hand [19].

Twenty-four orthoses with open elliptical pockets were 3D-printed on a Creality Ender 3 Pro 3D printer (Shenzhen Creality 3D Technology, Shenzhen, China) using red PLA filament (Devil Design Sp. J., Mikołów, Poland) and gray PETG filament (Devil Design Sp. J., Poland), with the following main process parameters (the other parameters, not listed below, had the default values for Cura Ultimaker 5.3.0 slicing software):

- Layer thickness: 0.2 mm
- Printing temperature: 215 °C (PLA), 230 °C (PETG)
- Platform temperature: 50 °C (PLA), 85 °C (PETG)
- Infill pattern: grid
- Perimeters: 2
- Top/bottom layers: 2
- Infill density: 55%, 80%
- Printing speed: 100 mm/s for layers, 60 mm/s for perimeters

A green PLA full orthosis (without open pockets) was also produced at 80% infill density.

The thermoforming of the 3DP-WHOs was conducted after warming the flat orthoses in hot water at temperatures exceeding the Tg for each material (85 °C for the PLA orthoses and 100 °C for the PETG orthoses). Then, the flat 3DP-WHOs were molded into a replica of the user’s wrist–forearm to obtain orthoses with a similar shape.

To determine the mechanical characteristics required for the subsequent use in the numerical modeling and simulations, PLA and PETG tensile specimens were modeled according to the ASTM D638 standard [30] and manufactured using the same filaments and printing parameters as the orthoses.

### 2.2. Finite Element Analysis of 3DP-WHOs

Two 3DP-WHOs (one with open pockets and one full/without open pockets) were 3D scanned after thermoforming using an Artec Eva 3D scanner (Artec 3D, Senningerberg, Luxemburg), these digital models being further used in the study. The full orthosis model was used not only for the moldability assessment but also as a reference for evaluating the impact of mass reduction achieved by designing open pockets, which provide usability benefits such as improved hygiene and ventilation, on the flexural strength of the orthosis.

The numerical model was developed in close alignment with the experimental model, aiming to validate the numerical analysis method using finite element method (FEM) software (ANSYS R2 2022). The objective of the numerical analysis conducted in ANSYS (Ansys, Inc., Canonsburg, PA, USA) was to determine the force value reached within the structure under imposed displacement. The model was experimentally validated, thus proposing this approach for use in other orthosis designs.

**Figure 2:**
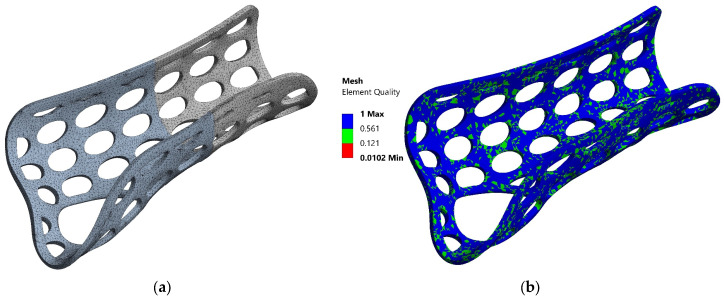
Discretization of the orthosis model with elliptical pockets (**a**). Mesh quality analysis (**b**).

The geometric model, obtained by 3D scanning the actual 3DP-WHO models formed through thermoforming, was imported into ANSYS. It was discretized (Figure 2a) using 2 mm-sized elements, resulting in a mesh with 159,007 nodes and 85,876 elements, conforming to the geometric features (such as geometric concentrators, fillet radii, etc.) present in the analyzed model. The mesh was modeled with finite elements of the type SOLID187. The element quality is presented in Figure 2b, ranging from 0.0102 (minimum) to 1 (maximum), with most elements showing good quality, as indicated by the dominance of blue and green colors.

**Figure 3:**
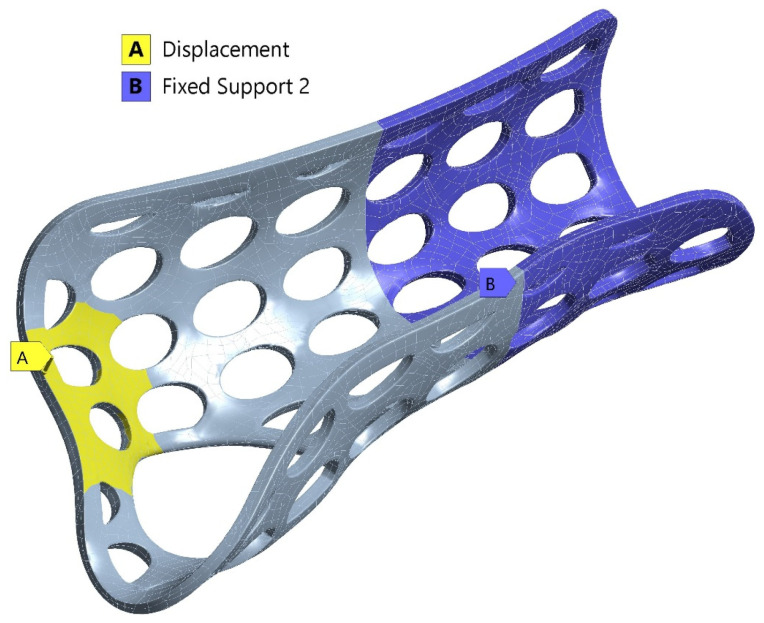
Boundary conditions showing the regions with displacement constraints (yellow) and fixed supports (blue).

After meshing, the boundary conditions were applied to simulate real-world constraints and expected deformations. Fixed supports were applied to specific regions, while displacement constraints were used to model the deflections observed during experimental testing (Figure 3). The displacement value at which the hand orthosis yielded during testing was used as the loading condition. Fixed support elements were applied across relevant areas, as shown in the experimental setup images (Section 2.3.2).

**Figure 4:**
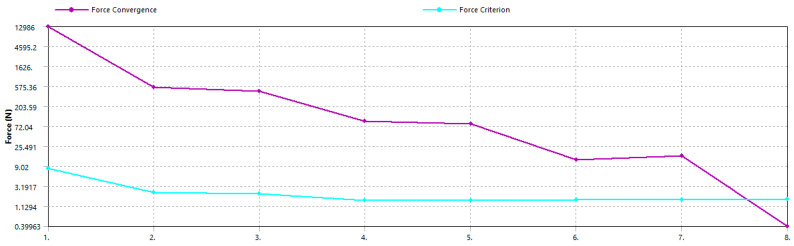
Force convergence plot showing the decrease in force residuals over iterations.

The analysis setup included defining the type of analysis (transient with nonlinear geometric effects) and the solution method (full Newton–Raphson). The solver computed the response of the structure iteratively, checking for convergence at each step. The solution converged after 219 iterations and 477,021 equations. The convergence criteria, focusing on force residuals, indicated effective convergence, as shown by the significant decrease in force residuals over the iterations in the convergence plot (Figure 4).

### 2.3. Experimental Tests

#### 2.3.1. Determination of PLA and PETG Material Properties

Digital Imaging Correlation (DIC) investigations were performed on PLA and PETG specimens to determine the mechanical characteristics required for FE simulations.

**Figure 5:**
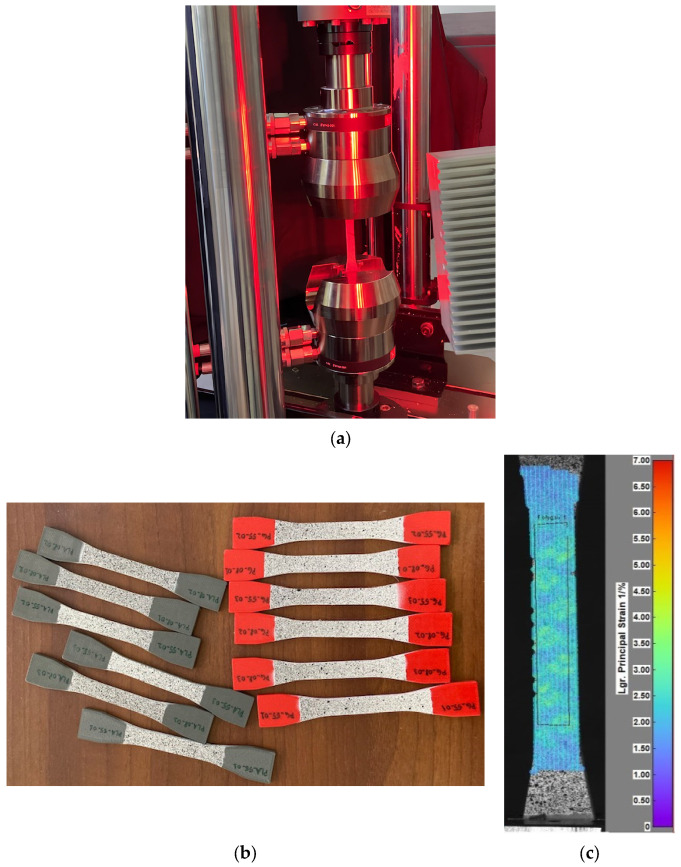
Experimental work: (**a**) image of tensile testing using DIC; (**b**) the gray PLA and red PETG specimens prepared for testing; and (**c**) illustrative image of DIC investigations, displaying a strain map with principal strain distribution on a tested specimen.

Batches of three specimens for each combination of material–infill were subjected to tensile tests (Figure 5a). For each sample, one of the specimen’s faces was spray painted with a coat of white and black speckle pattern (Figure 5b). The load was applied using an Instron 8872 (Instron Corp., Norwood, MA, USA) universal testing machine, with a loading speed of 1 mm/min, and strain reading was accomplished with a Dantec Dynamics DIC system (Dantec Dynamics Ltd., Tonsbakken, Denmark). Strain data were analyzed for a portion of the calibrated area (the polygon shape in Figure 5c), allowing the extraction of the characteristic curve, Young’s modulus, Poisson coefficient, apparent yield stress, and ultimate tensile stress.

#### 2.3.2. 3D-Printed PLA and PETG Orthoses Testing

**Figure 6:**
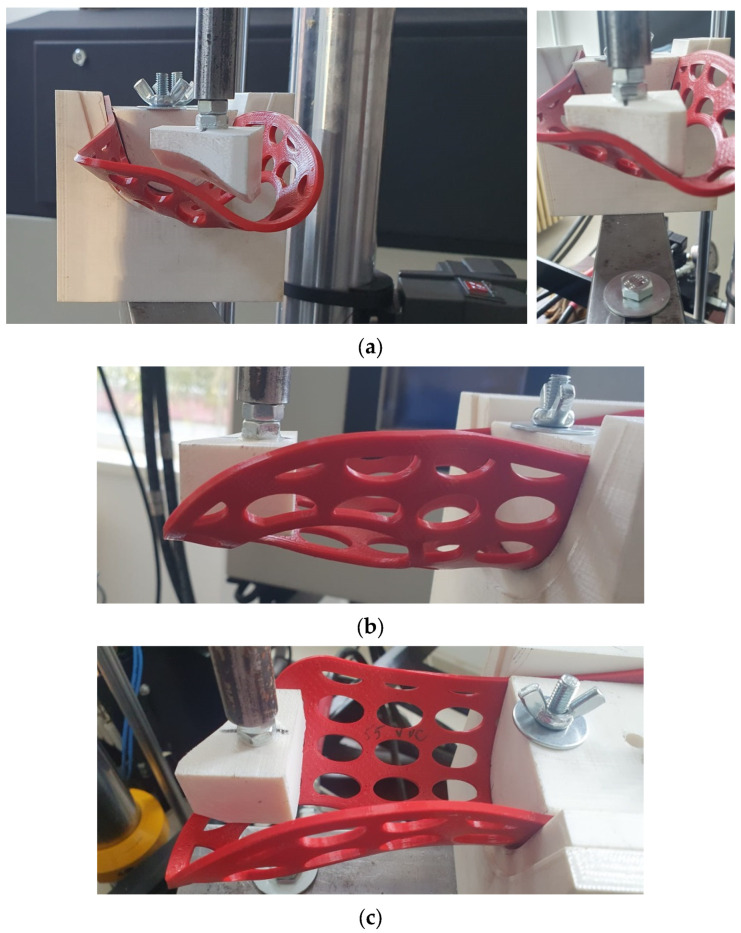
Three-dimensional printing wrist–hand orthoses flexural tests: (**a**) front views of the testing setup with force applied to the distal end of the PETG orthosis, (**b**) side view showing the orthosis bending, and (**c**) top view highlighting the orthosis fixation in the testing equipment and the zone where force was applied.

To investigate the mechanical resistance of the 3DP-WHOs subjected to flexion, 2-point static testing was conducted. Figure 3 shows the experimental setup on the same Instron 8872 UTM. The orthoses were fixed in a support made of ABS (Mojo 3D printer, Stratasys, Eden Prairie, MA, USA) in a cantilever position, as in the study by Cazon et al. [31] (Figure 6).

The force was applied to a surface at the distal end of the orthosis corresponding to the distal and proximal palmar creases, while the part of the orthosis usually fixed by Velcro straps on the forearm was immobilized in the support.

Quasi-static tests were performed on six specimens from each material–infill configuration. Three specimens of each configuration were tested immediately after manufacturing, while the other three were tested after being immersed in water and subjected to aging. Specifically, three PLA and three PETG orthoses were kept on a shelf for 13 months and immersed in water at 30 °C for 24 h prior to testing them using the experimental setup from Figure 6.

## 3. Results and Discussion

### 3.1. Moldability Evaluation of 3DP-WHO Materials

**Figure 7:**
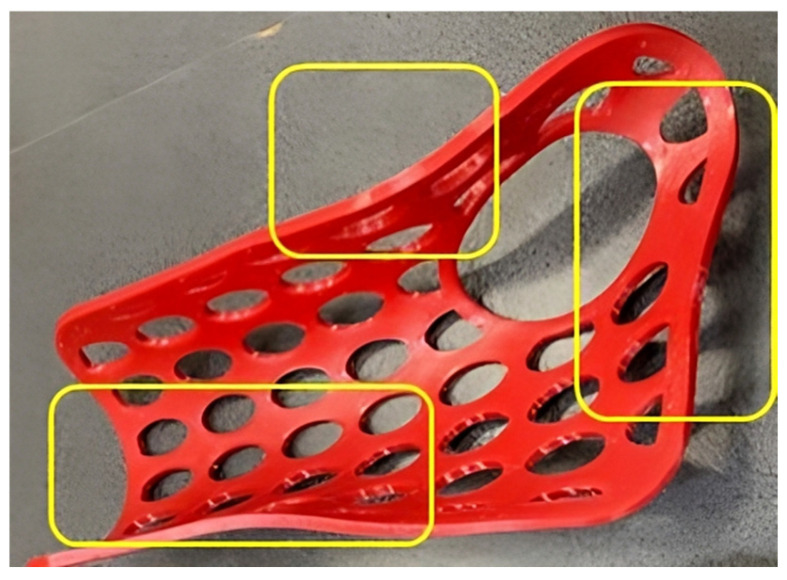
Highlighted zones of PETG orthoses that required remodeling for improved fitting.

Thermoforming the 3DP-WHOs made of PLA was easier than thermoforming the PETG counterparts. PETG orthoses not only required higher temperatures for softening and care in avoiding burning the patient hand, but they also became un-moldable very shortly after starting to cool down. The immersion time was set depending on the material and infill density. Thus, the 55% PLA orthoses were ready for thermoforming after 24 s, while the 80% PETG orthoses needed almost one minute until they became soft enough. The manually applied pressure for molding had to be maintained longer for the red PETG 3DP-WHOs because of their tendency to return to a previous form much faster than the PLA 3DP-WHOs. Moreover, the PETG orthoses did not properly follow the palm and wrist shapes, as did the PLA 3DP-WHOs, and they required localized reheating and remodeling onto the dummy hand–forearm (Figure 7). The 55% infill density orthoses proved more malleable compared to the 3DP-WHOs with 80% density, irrespective of the material used (PLA or PETG). As expected, the 55% infill density PLA 3DP-WHO without open pockets (the green orthosis in Figure 1b also made out of PLA took longer to heat in comparison to the open pocket PLA orthoses because of their closed-surface design). However, these observations were practical, and numerical investigations were conducted in [32] to better understand and simulate the orthoses thermoforming process.

The green PLA orthosis proved to be less moldable compared to its counterparts with open pockets. Its closed-surface design made the orthosis less flexible and thus more difficult to mold to the precise contours of the palm and wrist. This was theoretically expected and confirmed by practical experience. It is also worth mentioning that the PETG orthoses required three thermoforming processes to correctly fit the mold (forearm model), which we believe impacted the mechanical properties by acting as an annealing treatment on the material.

The printing time was 2 h 59 min for the 55% density orthoses and 3 h 20 min for the orthoses with 80% density. Summarizing, PLA’s lower softening temperature, faster moldability, and better conformity to the mold make it easier to thermoform 3D-printed orthoses compared to PETG. The lower infill density improved the malleability of orthoses from both materials.

### 3.2. Mechanical Tests Results

#### 3.2.1. Results of Mechanical Tests Performed on Specimens

**Figure 8:**
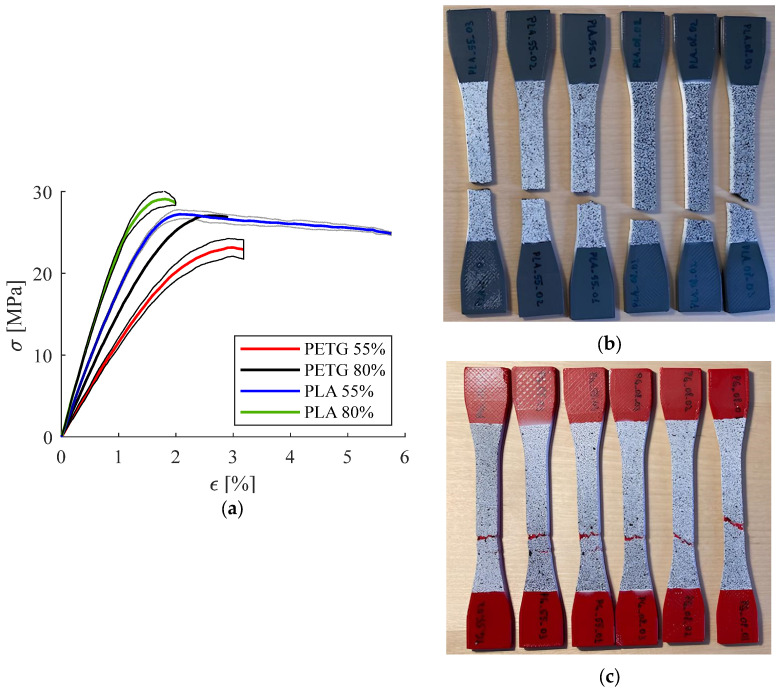
Experimental results for the PETG and PLA specimens: (**a**) stress–strain curves, (**b**) PLA specimens showing brittle fractures, and (**c**) PETG specimens showing ductile fractures.

Table 2 presents the results obtained for the tested PLA and PETG specimens. Figure 8a provides a graphical representation of these results. Three specimens were tested for each material–infill density combination (denoted PLA_55_01, PLA_55_02, and PLA_55_03, respectively, and PLA_80_01, PLA_80_02, and PLA_80_03).

These data were used further to conduct two-sample *t*-tests (using Microsoft Excel 2010) to compare PLA vs. PETG for each investigated property, as well as 55% vs. 80% infill density for each investigated property.

*t*-test results:

- Comparison between PLA and PETG: Significant differences (*p* < 0.5) were found in the Young’s modulus (*p* = 0.000480), Poisson coefficient (*p* = 0.020117), and apparent yield stress (*p* = 0.004521). However, when comparing the ultimate tensile strength (UTS), no significant difference was found (*p* = 0.430554).
- Comparison between 80% and 55% infill density:
  ○ PLA specimens: Significant differences were found in the Young’s modulus (*p* = 0.000121) and Poisson coefficient (*p* = 0.011542), but no significant differences were observed for the apparent yield stress (*p* = 0.090911) and UTS (*p* = 0.774743).
  ○ PETG specimens: Significant differences were found in all properties, particularly in the Young’s modulus (*p* = 0.006105), Poisson coefficient (*p* = 0.025838), apparent yield stress (*p* = 0.002054), and UTS (*p* = 0.017380).

As expected, a higher infill density resulted in a higher UTS and Young’s modulus. Comparing the mean values of the elasticity moduli, the PLA specimens recorded higher values than the PETG, confirming that PLA is a stiffer material compared to PETG [33]. This explains why molding pressure needed to be applied for a longer period for the PETG 3DP-WHOs and why it was more challenging to shape the PETG orthoses to follow the dummy hand form in the palm–wrist zone. The PLA specimens recorded brittle fractures irrespective of the infill density, while all PETG specimens failed in a ductile manner (Figure 8b,c). The highest Poisson ratio was recorded for the PETG with 55% infill density and the lowest for the 80% infill density PLA specimens. These observations are supported by the literature data [34,35].

It can also be noted that the mean UTS and the Poisson coefficient for the PETG samples with 80% infill density were similar with those of the PLA samples with 55% density. This is important, as a higher density means a longer printing time, and when setting the printing parameters, a trade-off between the printing time and cost and mechanical characteristics should be considered (the two materials have similar prices).

The statistical analysis results showed that PLA and PETG exhibit significant differences in mechanical properties, particularly in stiffness (Young’s modulus) and yield stress. Also, infill density impacts most mechanical properties, especially in PETG.

The results of the mechanical tests on the specimens were used further in the numerical analysis, with the tests performed on the thermoformed 3DP-WHOs made of PLA and PETG being used to validate the numerical model.

#### 3.2.2. Mechanical Tests Results Conducted on 3DP-WHOs

**Figure 9:**
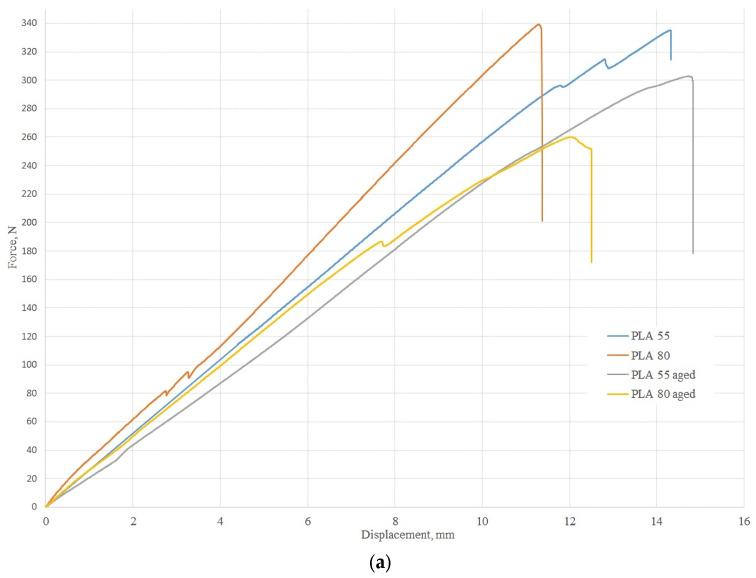

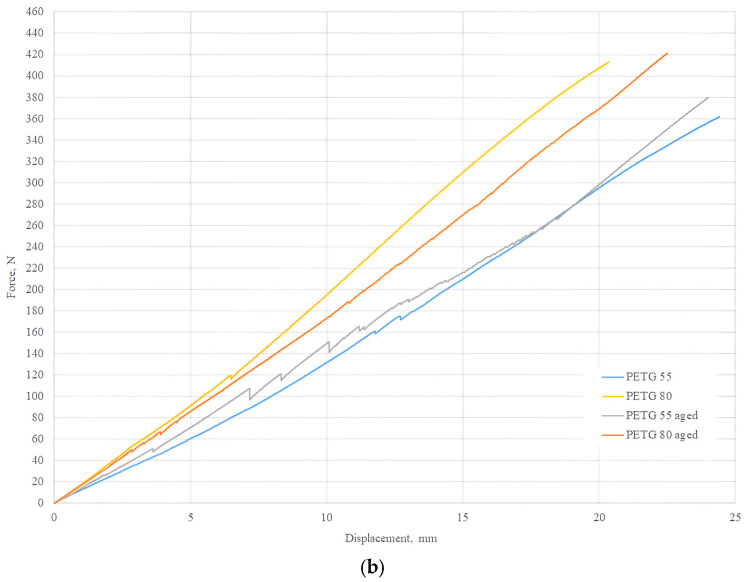
Three-dimensional printing wrist–hand orthoses flexural test results: (**a**) stress–strain mean curves for the PLA orthoses with 55% and 80% infill densities tested after 3D printing and after aging; (**b**) stress–strain mean curves for the PETG orthoses with 55% and 80% infill densities tested after 3D printing and after aging.

Figure 9 presents the mean stress–strain curves for the two-point flexural tests conducted on 3DP-WHOs made of PLA and PETG with two infill densities: 55% and 80%. In example, the curves are denoted as PLA 55 (for the PLA orthoses with 55% density) and PLA 55 aged (for the PLA orthoses with 55% density after aging).

Analyzing the graphics in Figure 9a for the PLA orthoses, it can be noted that the orthoses with 80% infill density failed at lower displacements. Additionally, the effects of moisture and aging were less significant for the 55% dense orthoses compared to their counterparts with 80% density.

From Figure 9b, it was noted that the effects of moisture and aging were not significant for the PETG 55% infill orthoses, which is an interesting behavior not reported so far in the literature. Possible explanations could include thermoforming, which can be assimilated to the annealing treatment [36], as well as stress relaxation and crosslinking over time, influencing the behavior of the PETG material, which is more elastic than PLA. PETG is a thermoplastic material that undergoes a curing process during 3DP that involves the formation of crosslinks between polymer chains. It might be that, over time, these crosslinks can continue to form and strengthen, leading to improved mechanical properties.

**Figure 10:**
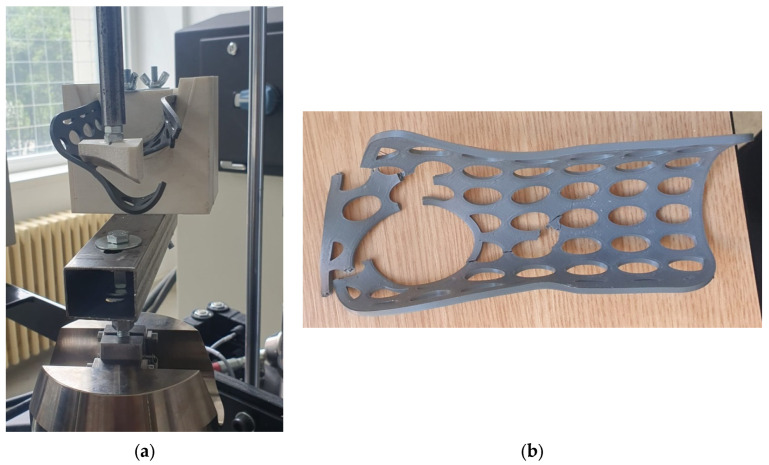
An example of a broken PLA during testing (**a**) and after being removed from the experimental stand (**b**).

The 3D-printed orthoses subjected to the combined effects of moisture and aging are compared with counterpart orthoses tested immediately after manufacturing. The findings showed that the mechanical properties of the orthoses decreased after aging, as expected, but only for the PLA orthoses. For the PETG orthoses, moisture and aging improved the mechanical properties. Additionally, the PETG orthoses, both initial and aged, did not break during the tests, confirming better elasticity in comparison to the PLA orthoses, whereas the PLA orthoses broke during the tests in a brittle manner (Figure 10).

Analyzing the behavior of aged PETG orthoses based on prior data from the literature, a decrease in the tensile strength was noticed for the PETG samples subjected to accelerated aging [28]. Also, Sedlak et al. investigated 3D-printed specimens made from different materials (ABS, PLA, PETG, and ASA) to understand the effect of various degradation factors on their mechanical behavior. Their conclusion was that PETG performs the best among the tested materials, but its properties still degrade with exposure to humidity and UV [26]. However, there are also studies indicating an increase in the flexural properties with annealing [36] and aging [37], but the majority of the data showed decreasing properties. In this context, our findings improved.

Banjo et al. [38] showed a significant degradation of the PLA mechanical properties after immersion in water at 70 °C for more than seven consecutive days. For less than a week immersion at 21 °C water temperature, the strength’s reduction was not relevant. UVB exposure for 24 h determined a decrease by 5.3% of the tensile strength for PLA [27]. However, the decrease in stiffness of the PLA specimens was not statistically significant. The moisture and UV aging combined effect has not been studied before, these conditions being relevant while using the 3DP-WHOs while washing or exposing the forearm wearing the orthosis to ultraviolet outdoor radiation.

For selecting the optimal material for a spinal brace application, a recently published paper studied PLA and PETG [33] from two perspectives: mechanical properties and finishing (PETG was found superior). However, Ronca et al. conducted experiments on specimens, not on the product, and announced further investigations for optimizing the process parameters [33]. A short communication on the material selection for 3D-printed casts was also found during the literature analysis [39], proving the practical relevance of such an analysis. Three-point bending tests were performed on diverse polymers, including perforated specimens of PLA, PETG, ABS (acrylonitrile butadiene styrene), and PC (polycarbonate) with different infill densities. However, the research methodology applied in [39] did not providing enough data for a proper and useful comparison. Another study by Schlégl et al. [40] compared fiberglass and traditional plaster casts with 3DP-WHOs made of PLA and PLA-CaCO_3_. First, the materials were mechanically characterized, and their water absorption was assessed. The results showed that PLA material performs better from all the analyzed aspects. Tests were performed on specimens, the orthoses being used only to examine and compare their surface qualities.

### 3.3. FEA Results

**Figure 11:**
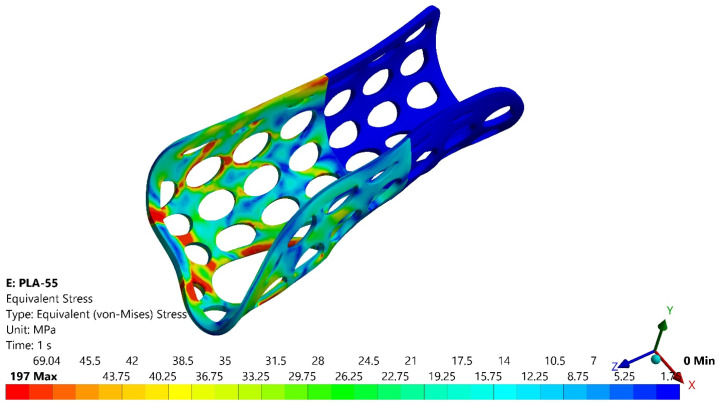
Variations of the equivalent stress for the PLA orthosis with 55% infill density.

**Figure 12:**
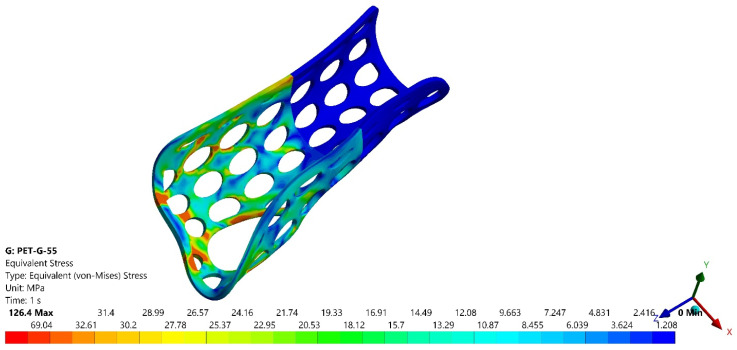
Variations of the equivalent stress for the PETG orthosis with 55% infill density.

Figure 11 and Figure 12 present the variations of equivalent stress (von Mises criterion) for two of the four analyzed models, namely PLA 55 and PETG 55.

It could be seen that the most stressed zone corresponded to the fractured zones from the experimental tests, as presented in Figure 10 for the PLA orthosis with 55% infill density.

In Table 3 are presented the forces corresponding to a 10-mm displacement for the PLA and PETG orthoses with 55% and 80% infill densities in the first set of experiments (F_EXP_) and those obtained by numerical simulation (F_FEA_), which proves the numerical model validation for each of the two infill densities and studied materials.

A similar numerical analysis conducted on the green PLA orthosis showed a maximum von Mises stress of 432 MP, compared to approximately 303 MPa for the orthosis with pockets, highlighting the impact of the slim design on the mechanical strength. However, both the experimental and numerical results indicate that even the 55% infill density PLA orthosis is suitable for safe daily use.

Górski et al. [24] studied customized wrist–hand casts made of PLA, polyamide 12, ABS, and HIPS (high-impact polystyrene) 3D-printed with different parameters (called economic, accurate, and strong modes). The orthoses were tested for three-point bending, and a FEM model was developed and validated. One of the conclusions of the research was that 3D-printed PLA casts in economic mode are suitable for most of the users. The studied orthoses were 3D-printed in different build orientations but directly in their end-use form, while, in our research, the focus was on thermoformed orthoses initially 3D-printed as flat.

Łukaszewski et al. [41] investigated different types of samples made of ABS as part of a 3DP-printed customized wrist–hand cast, as well as the whole cast. The samples were subjected to three-point bending tests for determining their modulus of elasticity, and the authors made a comparison with the corresponding numerical models (isotropic properties were assumed). The results showed that the value of the Young’s modulus for specimens like the middle part of the orthosis was similar to those experimentally determined for the whole orthosis.

In both these papers, 3D-printed full casts were subjected to three-point bending, while, in our research, a two-point bending testing was selected as corresponding to the tasks in which the user wearing a splint (not a full cast) is lifting a weight by performing flexion/extension movement. Cazon et al. [31], however, tested full multi-material splints made by the Polyjet process in flexion/extension and radial/ulnar deviation movements in a cantilever position. FE-based models of a 3D-printed splint and conventional splint were reconstructed from 3D scanning data. The traditional splint recorded maximum displacement in the metacarpal zone, while the 3D-printed splint showed no particular zone with maximum displacement.

## 4. Conclusions and Further Work

This study explored the mechanical performance of 3DP-WHOs made from PLA and PETG, focusing on the effects of thermoforming and infill density on their flexural properties.

The key conclusions of the investigations can be summarized as follows:

- Thermoformed 3D-printed orthoses made from both PLA and PETG demonstrated superior flexural resistance, even after being subjected to moisture and long-term aging. This shows that the thermoforming method can produce orthoses that are not only more easily customizable to the user’s hand and fulfil the immobilization functional criterion but also withstand aging and moisture. No such research has been conducted so far, thus opening up a new perspective for manufacturing orthotic products in 3DP-PoC.
- PLA orthoses were easier to thermoform, while the PETG material provided better elasticity and stability of the orthotic properties over time. Thermoforming was simpler for the PLA orthoses than PETG orthoses, requiring higher temperatures and faster cooling rates, complicating the molding process. After one year of shelf life and exposure to moisture, the PETG orthoses with 55% infill density showed properties almost identical to their initial status, likely due to thermoforming (acting as successive annealing), stress relaxation, and additional crosslinking over time. Conversely, the PLA orthoses experienced a more significant decrease in mechanical properties after aging, with the 55% dense orthoses being less affected than the 80% dense orthoses.
- The 3D-printed orthoses with 55% infill density can be produced as flat shapes easily, quickly, and with sufficient mechanical resistance. Given the challenges of 3D printing orthoses in their final, ready-to-use form at this infill density, the thermoforming approach provides significant advantages.
- The numerical and experimental analysis conducted in this study provides valuable insights into optimizing the material selection and design parameters, filling a gap in the existing literature.

This study presents promising future possibilities for medical orthotics. The successful thermoforming of flat 3D-printed orthoses offers a novel approach for creating customized patient-specific devices that maintain mechanical integrity during use. Using orthosis digital templates and thermoforming them can simplify the production process by reducing the need for extensive 3D scanning and modeling, as well as the fabrication time. The results of this study add more knowledge to the field, which is very important for gaining the trust of specialists and patients in using these medical devices.

Both PLA and PETG proved to be suitable materials for the application, even under degradation conditions like aging and moisture, suggesting that these materials are also good candidates for other types of orthotic devices, such as spinal braces or lower limb orthoses. Additionally, integrating 3D printing and thermoforming into healthcare facilities could facilitate the rapid, on-demand production of orthotic devices, enhancing patient care through immediate customization and adjustments.

## Figures and Tables

**Table 1:** SWOT diagram for 3D-printed customized orthoses.

	**3DP-WHOs Based on 3D Scanning**	**3DP-WHOs Based on Thermoforming**
**Strengths**	Enhanced customization to patient’s hand shape Improved fit and comfort	Efficient production process Cost-effective due to reduced material waste Faster production times Potential for scalable manufacturing and distribution Improved durability and reduced risk of breakage
	Ability to accommodate complex anatomical variations Ability to create complex designs that are challenging with traditional methods Enhanced patient engagement through personalized design Potential for improved functional outcomes and patient satisfaction Potential for reduced complications and re-hospitalizations	
**Weaknesses**	High initial investment in 3D scanning equipment Limited availability of advanced 3D scanning technology Requirement of specialized engineering knowledge to process the patient data and design the orthosis Potential for longer lead times compared to off-the-shelf solutions	Quality control challenges during thermoforming Limited material options for thermoforming Requirement of engineering knowledge to design the orthosis Potential for less precise fit compared to the 3D scanning-based approach
**Opportunities**	Increased adoption in healthcare Potential to speed up development of bespoke solutions for patients Collaboration with healthcare providers to refine the design and manufacturing process	
**Threads**	Regulatory issues for 3D-printed medical devices Reimbursement challenges for personalized 3D-printed orthotic solutions	

**Table 2:** Mechanical properties and statistical analysis of the PLA and PETG specimens.

Specimen	Young’s Modulus [MPa]	Poisson Coefficient	Apparent Yield Stress [MPa]	UTS [MPa]	Mean Young’s Modulus [MPa]	Mean Poisson Coefficient [-]	Mean Apparent Yield Stress [MPa]	Mean UTS [MPa]
		[-]						
PLA_80_01	2420	0.337	28.09	29.99	2384 ± 35.08	0.348 ± 0.01	27.3 ± 1.12	26.39 ± 5.14
PLA_80_02	2381	0.349	-	20.5				
PLA_80_03	2350	0.357	26.51	28.67				
PLA_55_01	1842	0.389	24.61	27.24	1864 ± 20.66	0.383 ± 0.01	24.4 ± 1.1	27.36 ± 0.57
PLA_55_02	1883	0.378	25.39	27.98				
PLA_55_03	1867	0.383	23.21	26.86				
PETG_80_01	1581	0.381	22.88	27.96	1538 ± 54.12	0.381 ± 0.0	22.86 ± 0.72	27.61 ± 0.32
PETG_80_02	1477	0.379	23.57	27.55				
PETG_80_03	1555	0.382	22.14	27.33				
PETG_55_01	1115	0.425	18.04	22.17	1190 ± 83.52	0.419 ± 0.01	18.52 ± 0.42	23.36 ± 1.14
PETG_55_02	1175	0.406	18.85	23.46				
PETG_55_03	1280	0.426	18.66	24.45				

**Table 3:** Comparative results, experimental vs. numerical.

Orthosis	PLA		PET-G	
	PLA 80	PLA 55	PETG 80	PETG 55
F_exp_ [N]	303.43	259.13	195.54	156.65
F_FEA_ [N]	319.26	250.25	206.45	160.18
Error [%]	5.21	3.42	5.57	2.25

## Data Availability

The original contributions presented in the study are included in the article, further inquiries can be directed to the corresponding authors.
